# Management of an Intracanal Separated Instrument in the Lower Right First Molar: A Case Report

**DOI:** 10.7759/cureus.63418

**Published:** 2024-06-28

**Authors:** Pratik Rathod, Aditya Patel, Anuja Ikhar, Manoj Chandak, Joyeeta Mahapatra, Tejas Suryawanshi, Jay Patil, Priti Mahale

**Affiliations:** 1 Department of Conservative Dentistry and Endodontics, Sharad Pawar Dental College and Hospital, Datta Meghe Institute of Higher Education and Research, Wardha, IND; 2 Department of Orthodontics and Dentofacial Orthopaedics, Annasaheb Chudaman Patil Memorial Dental College and Hospital, Maharashtra University of Health Sciences, Dhule, IND; 3 Department of Conservative Dentistry and Endodontics, Shri. Yashwantrao Chavan Memorial Medical and Rural Development Foundation's Dental College and Hospital, Maharashtra University of Health Sciences, Ahmednagar, IND

**Keywords:** broken instrument, separated instrument, endodontic treatment, ultrasonics, endodontics

## Abstract

Separating an endodontic instrument is one of the most frequent errors during a root canal treatment. If endodontic instruments get separated, it could hinder disinfection and prevent access to the apical portion of the root. It compromises the success of the treatment by impeding the proper debris removal from the canal. But now that techniques and tools have advanced, it is feasible to remove a separated instrument from the root canal successfully. This case report presents the management of a separated instrument, demonstrating the successful removal of the separated instrument.

## Introduction

The completeness of the cleaning and shaping processes determines how well a root canal treatment works. However, the presence of broken files poses a significant risk, as it impedes the canal's biomechanical preparation and obturation, ultimately resulting in treatment failure [1]. Endodontic instruments such as hands and rotary files are primarily made of stainless steel and nickel-titanium alloys, making them susceptible to breakage. Statistics show that the separation of instruments ranges from 1.68% to 2.4% for rotary instruments and approximately 0.25% for hand instruments [2]. The most common causes of file separation include improper use, physical limitations of the instruments, inadequate access, root canal anatomy, and potential manufacturing defects. The separated fragment can obstruct access to thorough root canal cleaning and shaping beyond the level of separation or irritate the periapex if it extends out of the root apex [1]. This is significant because it affects the final outcome of endodontic therapy. Therefore, an attempt should be made to bypass or retrieve the instrument before leaving it in place and obturating to the level of separation, or before considering surgical intervention [3].

Numerous alternative treatments exist for this scenario, such as bypassing it while it remains within the root canal or retrieving the separated fragment [1]. Handling such instances requires consideration of a number of parameters, such as visibility, the location of the separated fragment, and the remaining tooth structure [1]. A ledge may form, the file may be accidentally pushed farther apically, fragments may be extruded beyond the apex, excessive dentin removal may fracture the tooth, and root perforation may occur. These difficulties make the treatment more complicated and frequently require specialized assistance [3].

New tools for file retrieval have been made possible by technological advancements, such as specialty pliers (Steiglitz forceps, Hu-Friedy, Chicago, Illinois), microtube devices (Masserann microtubes, Micro-Mega, Besancon, France), and ultrasonic probes (Start X, Dentsply, Charlotte, North Carolina). These tools are often used in conjunction with microscopes to enhance visual clarity during the process. This case report shows the use of an ultrasonic device in conjunction with a microscope to increase precision for the retrieval of broken instruments.

## Case presentation

**Figure 1:**
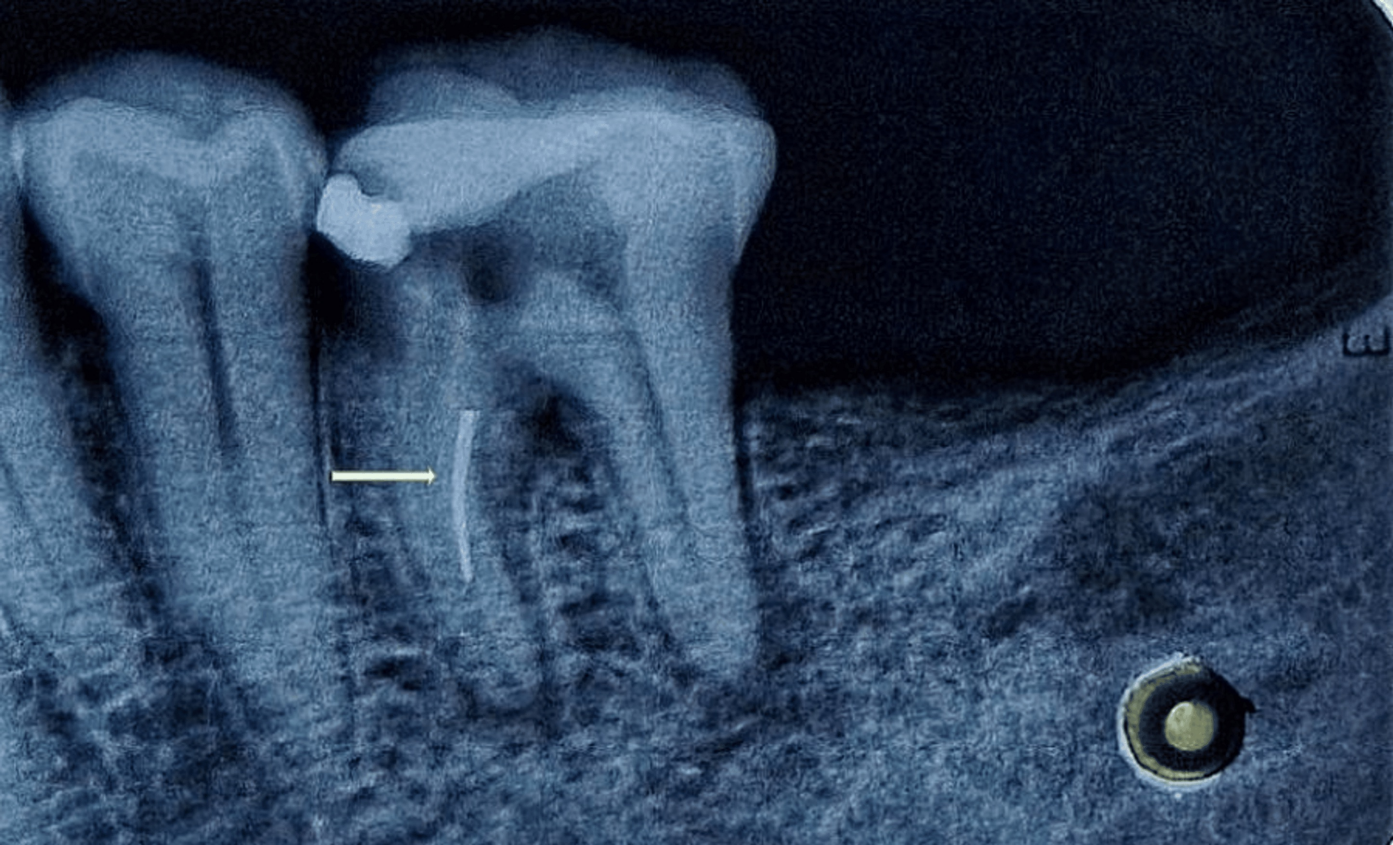
A separated instrument is seen in the mesiolingual canal The radiograph displays a separated endodontic instrument within the mesiolingual canal.

A 50-year-old woman reported to the Department of Conservative Dentistry and Endodontics with a complaint of discomfort while chewing in her lower right back teeth. Consequently, root canal therapy was performed a week ago in a private dental office following spontaneous pain for a year. During an intraoral examination, it was observed that caries had reached the pulp of an affected tooth. Subsequently, neurosensibility tests were performed, which showed negative vitality. There was a positive percussion test concerning the affected tooth. Radiolucent lesions were seen in the bifurcation region and at the apical regions of the distal and mesial roots, according to radiographic analysis. Additionally, a separated instrument was observed in the midsection of the mesiolingual root (Figure 1). Based on clinical and radiographical examinations, the diagnosis was a previously initiated therapy with symptomatic apical periodontitis. The nonvital root canals were the main aim of the treatment.

**Figure 2:**
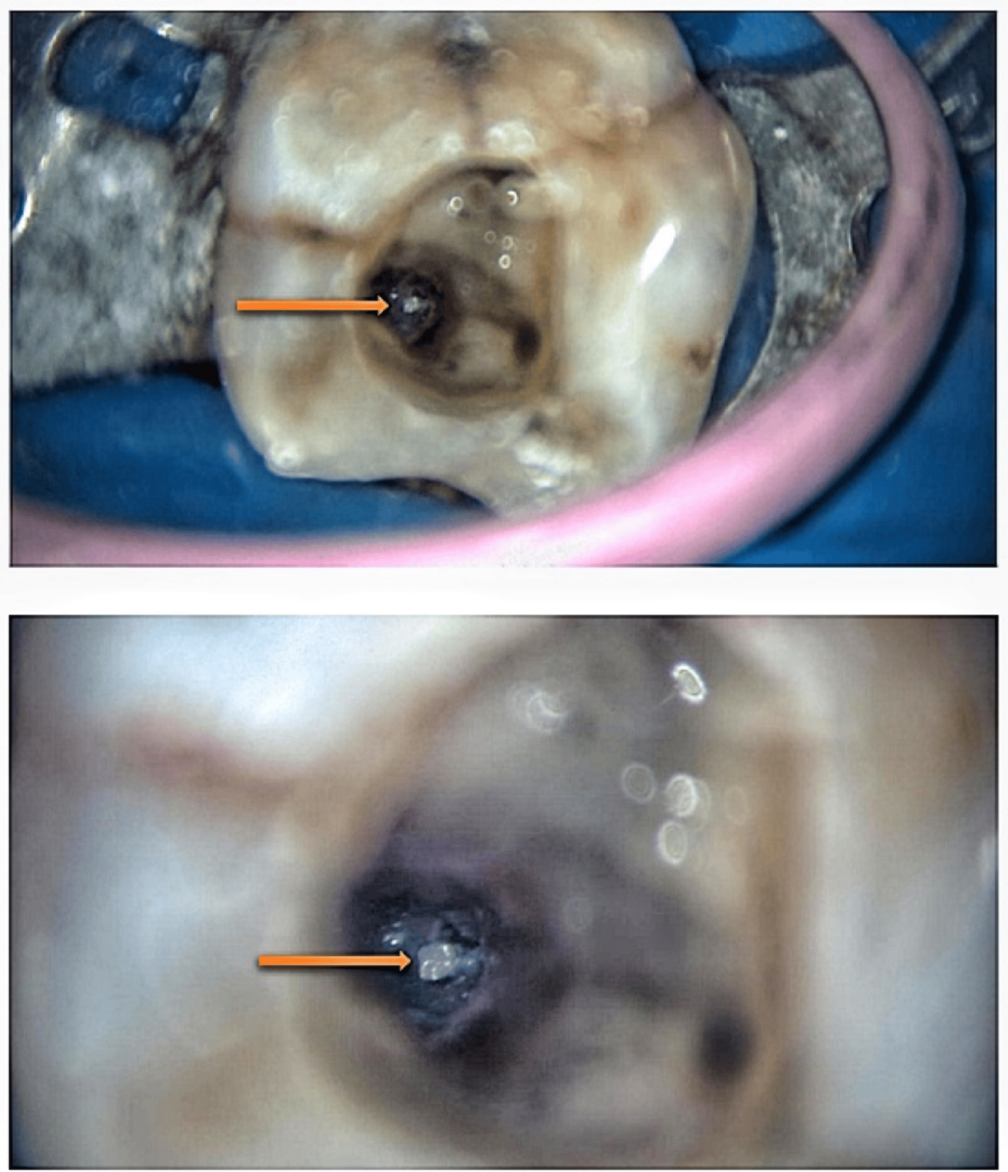
The separated instrument visible under the microscope The separated instrument is observed under magnification using a dental operating microscope.

The tooth was isolated using a rubber dam and prepared to provide sufficient coronal access under the dental operating microscope. After that, debris was removed using an irrigant containing 2.5% sodium hypochlorite (CanalPro, Coltene, Cuyahoga Falls, Ohio). The working length was established utilizing an electronic apex locator (Root ZX II, Morita, Irvine, California). The distal and mesiobuccal roots were shaped using Endo Plus 2019 rotary files (Guilin Woodpecker, Guilin, China) until the master apical file at W3 (0.06)/19 mm and W3 (0.06)/18 mm in the mesiobuccal and distal root, respectively. The root canals were irrigated, and Teflon and a paper point were used to close the openings to stop the entry of file fragments. To initiate the retrieval process for the mesial root canal, a staging platform was created using the X Gold ultrasonic tip (Eighteeth, Changzhou, China) until approximately 2-3 mm of the separated instrument was unveiled (Figure 2). The procedure aimed to loosen the file from the dentin's root canal wall to create space for the retrieval device. This space, known as the staging platform, is the gap between the root canal wall and the exposed file tip. The separated instrument was then maneuvered counterclockwise, generating an unscrewing force effect, which aids in the retrieval of the file through a clockwise cutting motion. The applied energy assists in loosening the separated instrument and creating a space between the wall of the root canal and the separated instrument. The X Blue ultrasonic tip could then be utilized to further loosen any fragments adhering to the wall.

**Figure 3:**
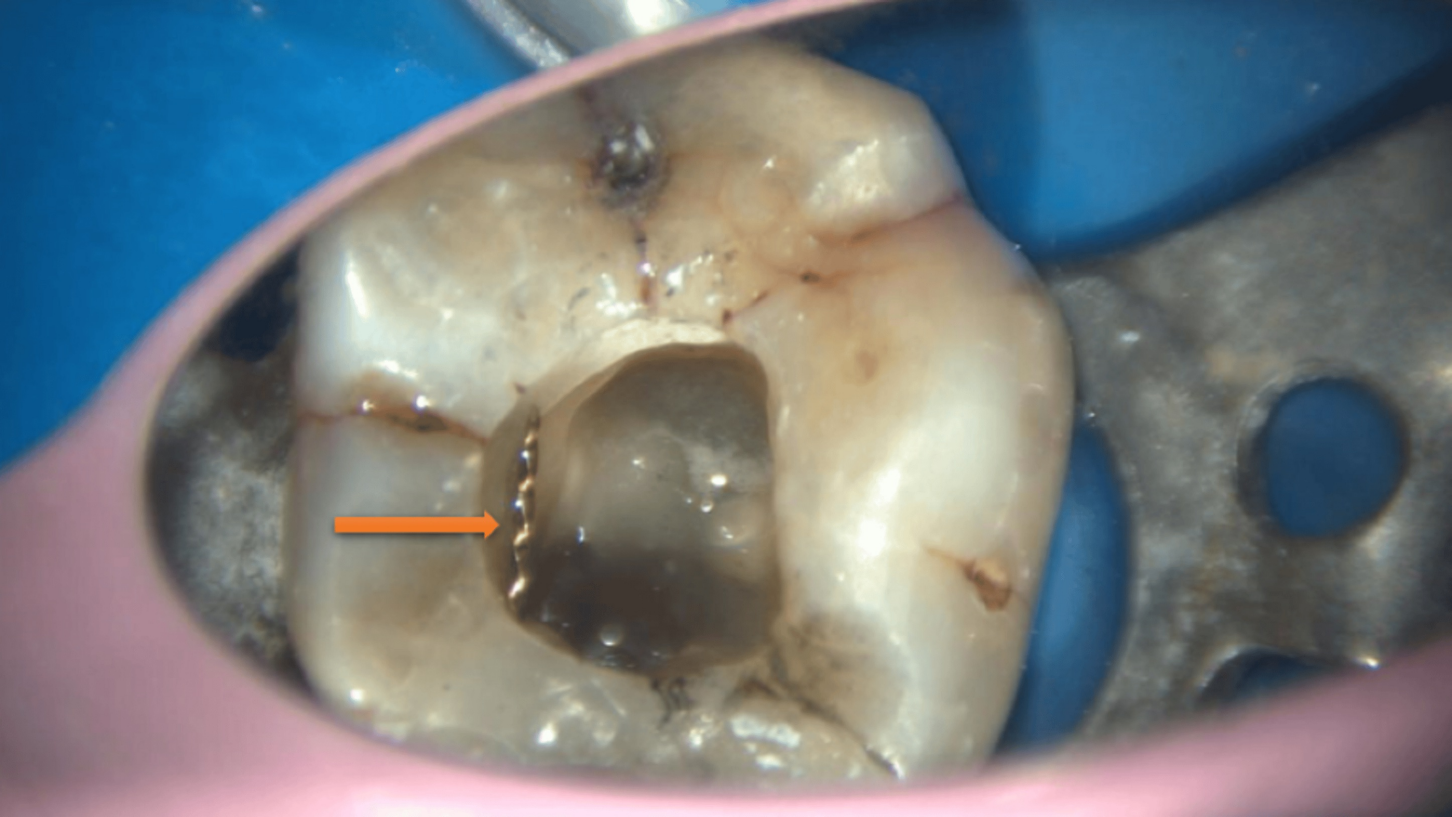
Successful retrieval of the separated instrument The retrieved, separated instrument is visible under magnification.

Irrigation was performed using 2.5% sodium hypochlorite and 17% ethylenediaminetetraacetic acid (NeoEDTA gel, Orikam, Changzhou, China), with activation carried out using an Ultra X ultrasonic activator. After approximately 20-30 minutes of activation, the fragment became loosened and was dislodged from the canal (Figure 3).

**Figure 4:**
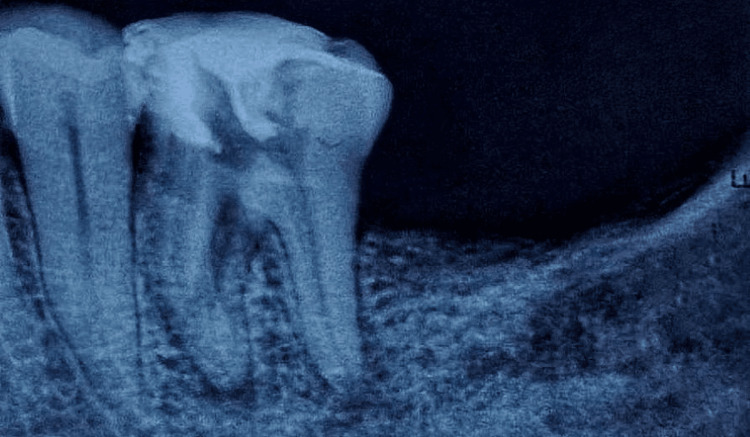
A radiograph showing the complete removal of the separated instrument The radiograph shows the complete removal of the separated instrument.

Following the successful removal of the file fragment, a radiograph was obtained to verify the complete retrieval of the fragment (Figure 4).

**Figure 5:**
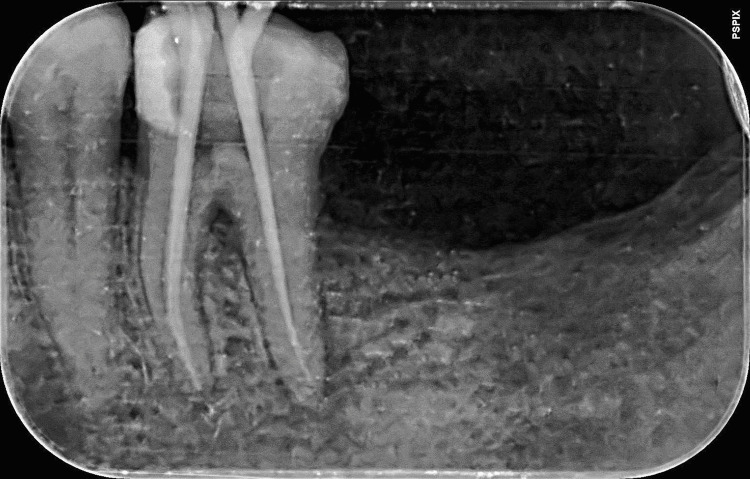
Master cone fit check A radiograph demonstrating a master cone fit check for the canal obturation procedure.

The mesiolingual root canal was shaped using Endo Plus 2019 rotary files up to W3 (0.06)/18 mm, with the appropriate master gutta-percha cone fit confirmed (Figure 5).

**Figure 6:**
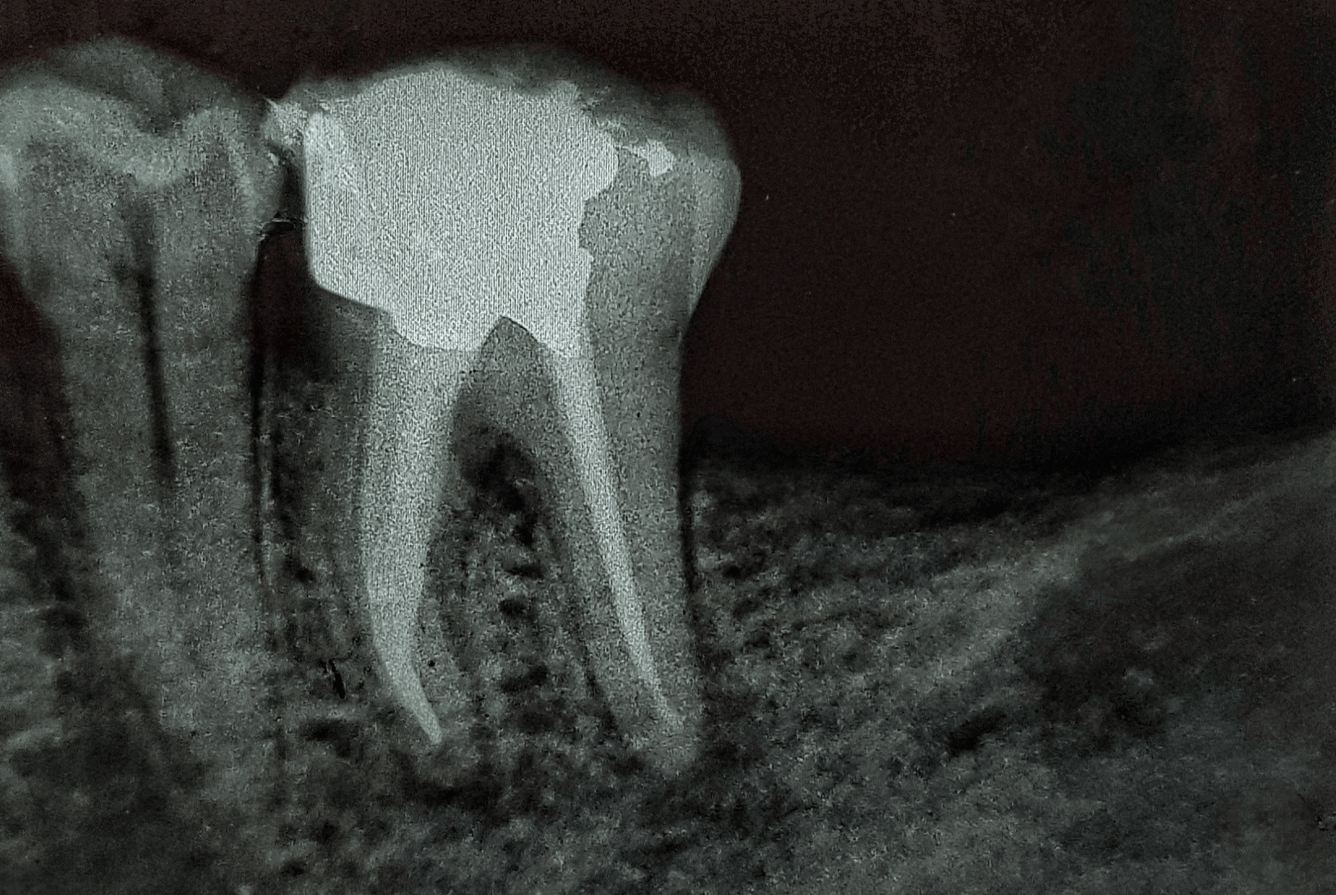
Post-obturation radiograph Postoperative radiograph after obturation and post-endodontic restoration of the treated tooth.

Calcium hydroxide paste (RC Cal, Prime Dental, Thane, India) was applied as medication, and the tooth was temporarily restored. The root canal was filled two weeks following the first appointment utilizing an MTA Fillapex sealer (Angelus, Londrina, Brazil) and gutta-percha (Dia-ProT, Diadent, Seoul, North Korea) in a continuous wave compaction technique. RMGIC (Fuji II LC, G.C., Oyama, Japan) and composite restoration (Spectrum, Dentsply, Charlotte, North Carolina) were used to seal the canal (Figure 6).

**Figure 7:**
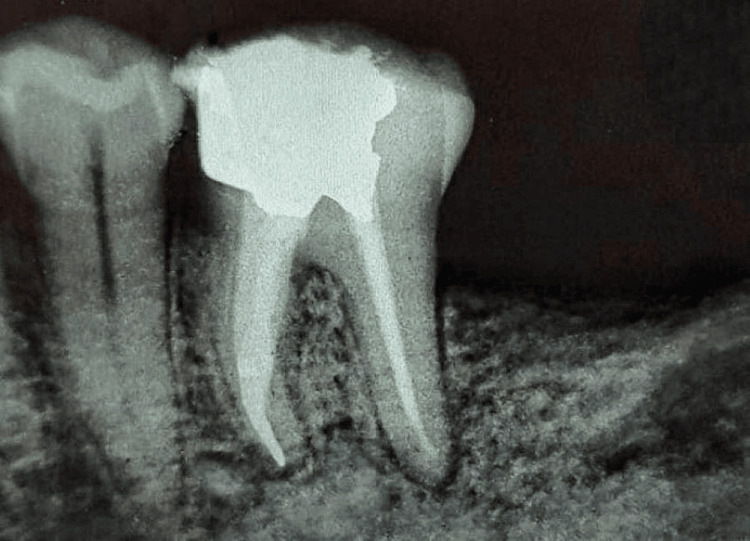
One-month follow-up radiograph A follow-up radiograph reveals the absence of any periapical lesion in the root canal-treated tooth, showing signs of healing.

**Figure 8:**
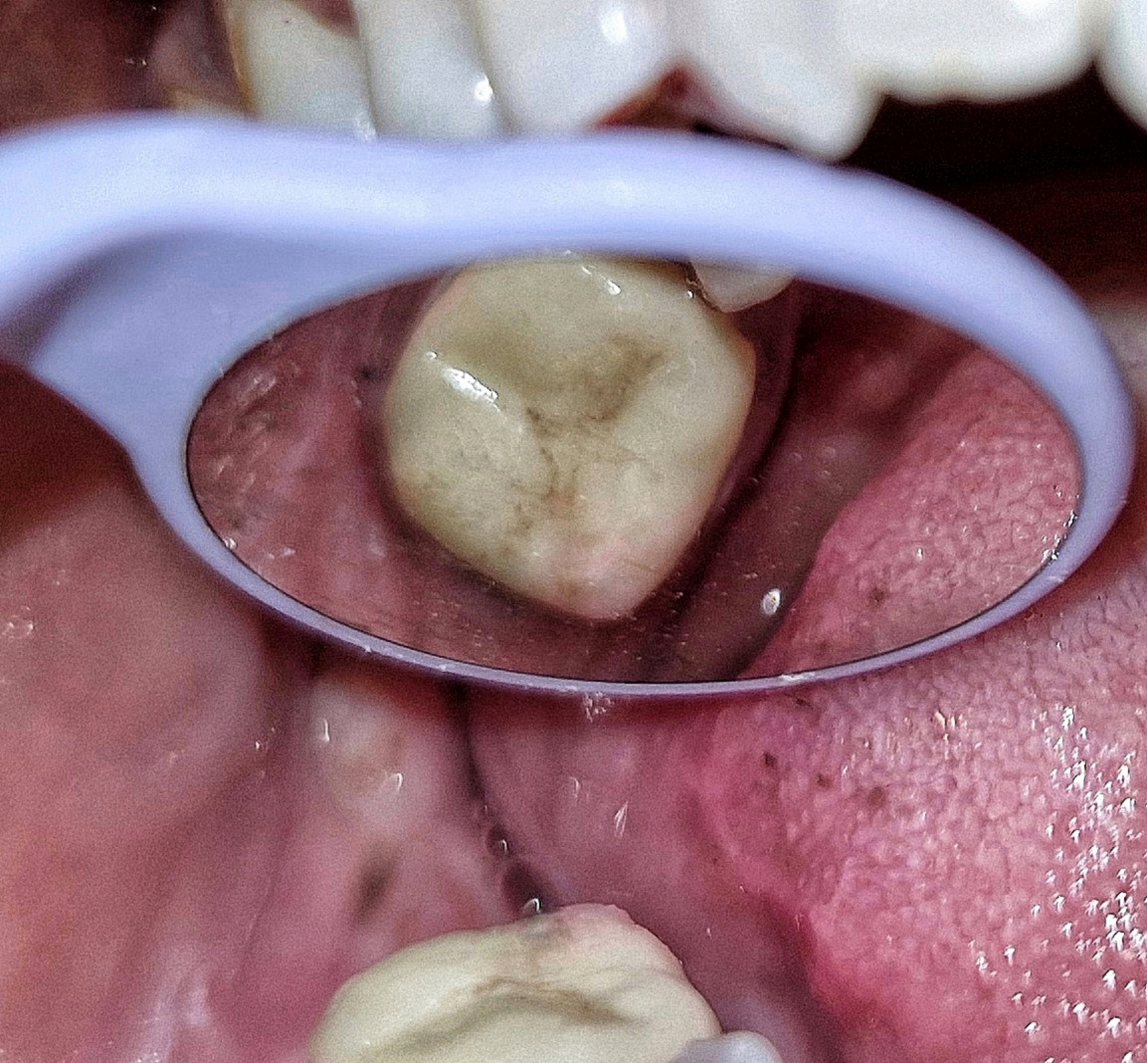
Clinical image after crown placement The image shows a cemented crown on the root canal-treated tooth.

After a month, the assessment revealed no significant results from the palpation, percussion, or subjective examination tests. Reduced apical radiolucency and bifurcation were seen in the one-month follow-up radiograph (Figure 7). After that, tooth preparation was done, and a crown was cemented using resin cement (Figure 8).

## Discussion

Utilizing an ultrasonic instrument under microscope assistance represents a conservative approach to managing a separated instrument, compared to alternative methods such as microtube extraction, microtube, and the glue method [4,5]. It is less likely to damage periodontal tissue and root integrity, although it can gently degrade dentin structure [5,6]. Drying out the area before doing the treatment allows for better sight under a microscope, which lowers the possibility of errors during the procedure [7]. However, there is unavoidable heat generation due to ultrasonic vibrations, and there is a chance that the temperature could increase by over 10 °C on the external root surface, which could potentially harm the periodontium [2]. If the ultrasonic tip comes into direct contact with a file, they are prone to secondary heat. As a result, EDTA irrigation was carried out with the ultrasonic tip set at the lowest power. This procedure improved the cleanliness of the root canal wall [2]. The tips that are being used are X Gold and X Blue from the Ultra X Retreatment Kit, which are made of an abrasive titanium niobium alloy. To create an unscrewing force effect on the file, the X Blue tip was applied in the middle one-third of the root canal, and the X Gold tip was rotated anticlockwise in the coronal one-third.

The continuous wave compaction technique was used to fill the root canal, and the canal was sealed with MTA Fillapex sealer, valued for its capacity to harden. MTA Fillapex offers numerous benefits, including promoting the formation of new tissue, mending damaged tissue rapidly without inducing inflammatory responses, possessing a high radiopacity that facilitates the interpretation of radiographs, and releasing calcium ions to hasten the healing of periapical lesions [8]. Additionally, it offers ease of insertion and handling, ample working time, and straightforward removal during retreatment, particularly when used with gutta-percha [7]. Due to its alkaline pH, MTA Fillapex exhibits antimicrobial activity against various microorganisms such as *S. aureus, P. aeruginosa, M. luteus, C. albicans, E. coli, P. aeruginosa, and E. faecalis *adding to its versatility and effectiveness in endodontic applications [9,10].

Advancements in technology and magnification aids have significantly improved the success rate of instrument retrieval in dental procedures. Using microscopes or magnifying loupes helps guide the retrieval process, reducing damage to the canal dentine. The use of ultrasonics in endodontics was first described by Richman in 1957. Initially, ultrasonic units operated at frequencies of 25-40 kHz. Later, ultrasonic handpieces were developed to operate at lower frequencies of 1-8 kHz, which produce less shear stress and cause minimal alteration to the canal surface. According to Nevares et al., the success rate of retrieving a visible fragment with a dental microscope is 85.5%, compared to a 47.7% success rate when the fragment is not visible [11]. In 2019, Pruthi et al. found a success rate of 90% in retrieving separated instruments using ultrasonics [12]. It should be noted that the magnification provided by the dental operating microscope significantly contributed to the success of ultrasonic instrument removal in the present case. According to Nagai et al. [13], the success rate of ultrasonic removal increased from 67% to 88% and 95% with the aid of dental microscope magnification.

In this case, a combination of ultrasonic tips was used under a microscope to improve visibility during the retrieval of the mandibular right first molar, exhibiting symptoms of apical periodontitis, and had a separated instrument in the mesiolingual root canal. Subjective complaints stopped after the treatment was completed, and the broken instrument was completely removed. Furthermore, the radiograph confirmed the positive result by showing a decrease in apical radiolucency.

## Conclusions

Advancements in technology, sophisticated tools, and increased expertise have enabled the successful management of fractured instruments. The ultrasonic technique provides a reliable method for retrieving separated instruments from the root canal while minimizing dentine loss.

## References

[1] Shenoy A, Mandava P, Bolla N, Vemuri S (2014). A novel technique for removal of broken instrument from root canal in mandibular second molar. Indian J Dent Res.

[2] Cohen S, Hargreaves K (2016). Pathways of the Pulp 11th Edition. Pathway’s of the pulp-11th edition.

[3] Lambrianidis T (2018). Management of fractured endodontic instruments. https://link.springer.com/book/10.1007/978-3-319-60651-4.

[4] McGuigan MB, Louca C, Duncan HF (2013). Clinical decision-making after endodontic instrument fracture. Br Dent J.

[5] Kwak SW, Shen Y, Liu H, Kim HC, Haapasalo M (2022). Torque generation of the endodontic instruments: a narrative review. Materials (Basel).

[6] Kwak SW, Shen Y, Liu H, Wang Z, Kim HC, Haapasalo M (2021). Heat treatment and surface treatment of nickel-titanium endodontic instruments. Front Dent Med.

[7] Gluskin AH, Ruddle CJ, Zinman EJ (2005). Thermal injury through intraradicular heat transfer using ultrasonic devices: precautions and practical preventive strategies. J Am Dent Assoc.

[8] Banerjee S, Sharma R, Roy P (2017). Bypassing a broken instrument in a severely curved root canal: a case report. Indian J Conserv Endod.

[9] Radeva E (2017). Bypassing a broken instruments (clinical cases). Int J Sci Res.

[10] Parashos P (2018). Prognosis of root canal treatment with retained instrument fragment. Management of Fractured Endodontic Instruments.

[11] Nevares G, Cunha RS, Zuolo ML, Bueno CE (2012). Success rates for removing or bypassing fractured instruments: a prospective clinical study. J Endod.

[12] Pruthi PJ, Nawal RR, Talwar S, Verma M (2020). Comparative evaluation of the effectiveness of ultrasonic tips versus the Terauchi file retrieval kit for the removal of separated endodontic instruments. Restor Dent Endod.

[13] Nagai O, Tani N, Kayaba Y, Kodama S, Osada T (1986). Ultrasonic removal of broken instruments in root canals. Int Endod J.

